# Clinical Applications of Cardiac Magnetic Resonance Parametric Mapping

**DOI:** 10.3390/diagnostics14161816

**Published:** 2024-08-20

**Authors:** Daniele Muser, Anwar A. Chahal, Joseph B. Selvanayagam, Gaetano Nucifora

**Affiliations:** 1 Cardiac Electrophysiology Unit, Department of Biomedical Sciences, Humanitas University, 20090 Milan, Italy; daniele.muser@gmail.com; 2 Cardiac Electrophysiology, Cardiovascular Medicine Division, Hospital of the University of Pennsylvania, Philadelphia, PA 19104, USA; 3 Center for Inherited Cardiovascular Diseases, WellSpan Health, Lancaster, PA 17601, USA; chahal.anwar@mayo.edu; 4 Barts Heart Centre, St Bartholomew’s Hospital, Barts Health NHS Trust, West Smithfield, London E1 1BB, UK; 5 Department of Cardiovascular Medicine, Mayo Clinic, Rochester, MN 55905, USA; 6 Department of Cardiovascular Medicine, Flinders Medical Centre, Adelaide, SA 5042, Australia; joseph.selva@sa.gov.au; 7 Cardiac Imaging Unit, NorthWest Heart Centre, Manchester University NHS Foundation Trust, Manchester M13 9WL, UK; 8 Division of Cardiovascular Sciences, School of Medical Sciences, Faculty of Biology, Medicine and Health, Manchester Academic Health Science Centre, University of Manchester, Oxford Road, Manchester M13 9PL, UK

**Keywords:** cardiovascular magnetic resonance, myocardial tissue characterization, parametric mapping, T1 mapping, T2 mapping

## Abstract

Cardiovascular magnetic resonance (CMR) imaging is widely regarded as the gold-standard technique for myocardial tissue characterization, allowing for the detection of structural abnormalities such as myocardial fatty replacement, myocardial edema, myocardial necrosis, and/or fibrosis. Historically, the identification of abnormal myocardial regions relied on variations in tissue signal intensity, often necessitating the use of exogenous contrast agents. However, over the past two decades, innovative parametric mapping techniques have emerged, enabling the direct quantitative assessment of tissue magnetic resonance (MR) properties on a voxel-by-voxel basis. These mapping techniques offer significant advantages by providing comprehensive and precise information that can be translated into color-coded maps, facilitating the identification of subtle or diffuse myocardial abnormalities. As unlikely conventional methods, these techniques do not require a substantial amount of structurally altered tissue to be visually identifiable as an area of abnormal signal intensity, eliminating the reliance on contrast agents. Moreover, these parametric mapping techniques, such as T1, T2, and T2* mapping, have transitioned from being primarily research tools to becoming valuable assets in the clinical diagnosis and risk stratification of various cardiac disorders. In this review, we aim to elucidate the underlying physical principles of CMR parametric mapping, explore its current clinical applications, address potential pitfalls, and outline future directions for research and development in this field.

## 1. Introduction

Cardiovascular magnetic resonance (CMR) stands as an advanced imaging technique that enables the non-invasive evaluation of the heart, devoid of ionizing radiation. It has earned recognition as the gold standard for assessing cardiac anatomy, structure, and function. One of the transformational strengths of CMR lies in its capacity to discern soft-tissue characteristics, setting it apart from alternative imaging methods. This capability allows CMR to provide insights into the existence of fatty infiltration, inflammatory edema, and necrosis/fibrosis, enhancing its diagnostic prowess. The possibility to detect areas of abnormal myocardium is qualitative and relies on the spatial heterogeneity of signal intensity (i.e., difference in signal intensity between abnormal and normal adjacent myocardium) to generate image contrast. The use of contrast agents in late gadolinium enhancement (LGE) imaging can increase contrast resolution due to different wash-in and wash-out kinetics of gadolinium in normal myocardium and in areas of replacement scar tissue. LGE performs best in cases where there are regional differences in tissue composition, for example in coronary-artery-disease-related myocardial oedema, necrosis, and/or scarring. By contrast, it performs less well in non-ischemic heart diseases, as collagen deposition is often diffuse in these conditions, as one is unable to ‘null’ normal myocardium [1]. For the same reason, LGE may fail to show significant abnormality in some cases of myocardial infiltrative disorders, such as Fabry disease, cardiac amyloidosis, or iron deposition cardiomyopathy, until advanced stages of the disease. In the last two decades, CMR parametric mapping techniques able to quantify tissue properties on the response to the magnetic field (i.e., T1, T2, T2* mapping) have progressively emerged as able to overcome the aforementioned limitations and the need for contrast agents [2]. Tissue MR properties are quantified on a single-voxel basis and are used to generate color-coded spatial myocardial maps, providing both quantitative and qualitative information that, compared to conventional T1- and T2-weighted methods, do not rely on relative image signal intensity to identify structural abnormalities.

In this narrative, contemporary review, we comprehensively address the basic physical principles behind CMR parametric mapping, its challenges, current clinical applications, and future perspectives.

## 2. Physical Principles

Parametric CMR mapping is a quantitative technique providing the absolute value of the relaxation time of each single voxel expressed in milliseconds. Relaxation times depend on tissue composition and the surrounding environment; thus, they can be used to identify abnormal tissue characteristics via comparison to predetermined normal values.

**Figure 1:**
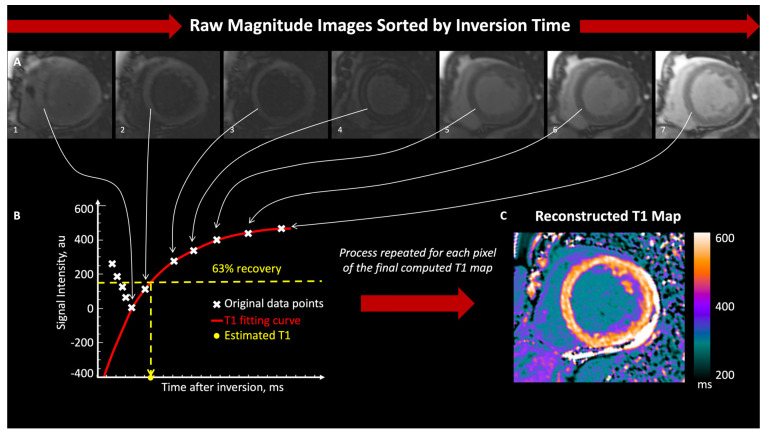
Example of Construction of a T1-Map. Myocardial T1-mapping using the Modified Look-Locker sequence with increasing inversion times (**A**). Multiple phases are acquired after an inversion pulse, then the software automatically determines pixel by pixel the signal intensity of each phase-image and subsequently plot it against the inversion time (**B**). Finally, the T1-time is automatically calculated by performing a curve fitting of the data points to an exponential recovery curve (**B**: red curve) representing the recovery of longitudinal magnetization. The T1-time is the constant of the exponential curve, represents the time to reach approximately 63% of longitudinal magnetization recovery and is used to generate a color-coded map (**C**). Reproduced with permission from Muser et al. [3].

After an electromagnetic inversion pulse perturbates nuclear spins, magnetization returns to the equilibrium following an exponential process, which has a time constant called relaxation time, representing the time to reach approximately 63% of magnetization recovery. Relaxation times can be calculated by acquiring a series of images at different time points after an inversion pulse; then, the pixel-by-pixel signal intensity of each phase-image is measured and plotted against the inversion time. Finally, the relaxation time is calculated by performing curve fitting of the data points to an exponential recovery curve and used to generate a color-coded map (Figure 1). In order to obtain an accurate parametric map, the physical displacement between the images in the series should be negligible, making cardiac parametric mapping particularly challenging in cases affected by both cardiac and respiratory motion. Respiratory motion is usually averted by breath holding while cardiac motion is avoided by limiting the acquisition window to a sufficiently short time at mid-systole or end-diastole. Any image shift can be potentially corrected through post-processing registration before pixel-by-pixel fitting [2].

## 3. T1 and T2 Mapping Imaging

Spin-lattice or T1 relaxation time is the time constant of recovery of longitudinal magnetization. T1 relaxation time primarily depends on the tissue composition itself (molecular environment of the water molecules in the tissue) but is also affected by several factors including heart rate, the specific technique used, the characteristics of the MR hardware (field strength), and environmental factors such as temperature and humidity [4,5]. For these reasons, T1 values may vary considerably between centers according to the MR hardware and specific pulse sequence design, making it difficult to establish an absolute range of normal values across centers. Hence, in both T1 and T2 mapping (see below), it is important that each center establishes its own specific normal reference values, with routine calibration, documenting reliability and validity, as well as inter- and intra-observer variability.

Several methods have been described to quantify myocardial T1 time, and most of them are based on inversion recovery (MOLLI: MOdified Look-Locker Inversion recovery; ShMOLLI: Shortened MOdified Look-Locker Inversion recovery; ANGIE: Acquisition of Non-Gradient Echoes; STONE: Spoiled Turbo Field Echo with Nonlinear Encoding), saturation recovery (AIR, SASHA, SAP-T1), or a mix of them (SAPPHIRE), with the modified Look-Locker inversion recovery method (MOLLI) and its variant, shortened MOLLI (ShMOLLI), being the most widely used [6,7,8,9].

The MOLLI sequence implies multiple intermittent (ECG-gated) acquisitions following a 180° inversion pulse repeated at different delay times. Specifically, a 3(3)3(3)5 scheme was initially adopted, in which three trains of three, three, and five raw images are acquired, separated by three pausing heart cycles to allow for signal recovery, resulting in a total of 11 raw images out of 17 total cardiac cycles [6]. The ShMOLLI scheme is a version of the MOLLI sequence, developed in order to shorten acquisition time and avoid the misregistration of raw images related to suboptimal breath holding. A total of seven raw images are acquired through nine heart beats, of which images six and seven are only considered for short T1 values to limit heart rate dependence. A further development to limit the influence of heart rate involves the acquisition of two trains of raw images for 5 s and 3 s, separated by a 3 s pause [5 s (3 s) 3 s] [10].

### 3.1. T1 Mapping

Native T1 mapping refers to the computation of T1 maps without the use of any contrast agent. Native myocardial T1 time depends on the specific tissue composition, which, in the case of myocardium, predominantly reflects myocyte characteristics but also the interstitial space and intravascular compartment. Therefore, native T1 times are highly sensitive to changes in tissue composition over a wide range of myocardial disorders. Higher T1 values are typically observed in cases of increase in free water content, such as myocardial inflammation and the expansion of extracellular volume (i.e., fibrosis deposition or amyloid infiltration) [11,12]. On the other side, native T1 values are reduced in cases of increases in fatty content (i.e., sphingolipids accumulation in Fabry disease) or iron overload [13,14]. The computation of T1 maps after the administration of gadolinium contrast agents, which typically shortens myocardial T1 times, fall under the name of post-contrast T1 mapping. Acquisition is typically performed 10 min after the administration of a contrast agent, but even at a fixed time point post-contrast administration, T1 times are affected by several factors, including body fat percentage and hematocrit and glomerular filtration rate, resulting in isolated post-contrast T1 maps of limited utility. The quantification of extracellular volume (ECV) fractions can overcome some of the limitations of post-contrast T1 mapping. ECV includes both interstitial and intravascular compartments and can be calculated starting from native T1 times, post-contrast T1 times, and the hematocrit, based upon the principle that gadolinium agents cause a shortening of T1 times proportional to their concentration in the extracellular space [15,16].

The ECV fraction of blood can be easily determined as

$${\mathrm{ECV}}_{\mathrm{blood}}=100-\mathrm{haematocrit}\;\left(\%\right)$$

Knowing the ECV fraction of blood and measuring T1 times in the blood before and after the administration of a contrast agent allows for the determination of the relationship between the ECV fraction and T1 times, and it consequently allows for the calculation of myocardial ECV as related to blood ECV by the following formula:

$${\mathrm{ECV}}_{\mathrm{myo}}=\frac{\frac{1}{T1\;\mathrm{myo}\;\mathrm{postGd}}-\frac{1}{T1\;\mathrm{myo}\;\mathrm{native}}}{\frac{1}{T1\;\mathrm{blood}\;\mathrm{postGd}}-\frac{1}{T1\;\mathrm{blood}\;\mathrm{native}}}\times \left(100-\mathrm{haematocrit}\right)$$

**Figure 2:**
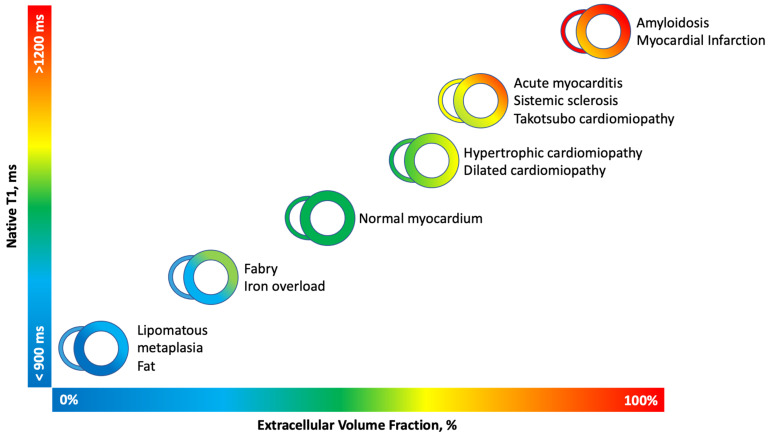
Native T1-mapping and Extracellular volume fraction in clinical practice. Native T1-mapping and extracellular volume fraction (ECV) have become valuable tools in clinical practice for tissue characterization. Native T1 and ECV offer insights into various pathological processes. However, it is important to acknowledge that the absolute values for native T1 are notably influenced by various factors, including field strength (1.5 T or 3 T), pulse sequence (MOLLI or ShMOLLI), and scanner manufacturer. To maintain clarity and consistency, this figure exclusively incorporates studies conducted using 1.5 T scanners. Figure adapted from Haaf et al. [18].

Unlike post-contrast T1 mapping values, ECV values are more reproducible and less affected by variables influencing isolated post-contrast T1 maps. Similarly, to native T1, the expansion of ECV is nonspecific and may be related to both the expansion of interstitial space, such as in myocardial inflammation or amyloid infiltration, and the expansion of intravascular content, such as in the case of acute myocardial infarction. The ECV fraction can be considered a surrogate marker of diffuse myocardial fibrosis when all the other confounding elements are ruled out. In cases in which the actual patient’s hematocrit value is not available, a ‘synthetic’ hematocrit value can be derived from the relationship between the change in blood T1 relaxation times pre- and post-contrast administration, given the linear relationship between hematocrit and blood T1 relaxivity. By accounting for the contrast agent’s dilution in the blood, the synthetic hematocrit compensates for any variability in the patient’s actual hematocrit level. Using the synthetic hematocrit enables the calculation of synthetic ECV, which has been demonstrated to be reliable both on 1.3T and 3T scanners (Figure 2) [17].

Pre-contrast T1 mapping provides valuable information about myocardial tissue characteristics, such as fibrosis and edema, without the need for contrast administration. It is often sufficient for routine clinical assessments and can detect abnormalities in various cardiac conditions. However, ECV measurement considers both pre- and post-contrast T1 values, providing a more comprehensive assessment of myocardial fibrosis and interstitial changes. ECV quantification is particularly useful in conditions where there is a significant alteration in extracellular volume, such as diffuse fibrosis or infiltrative diseases like amyloidosis. In such cases, ECV offers additional diagnostic and prognostic insights beyond native T1 mapping alone.

### 3.2. T2 Mapping

Spin–spin or T2 relaxation time is a fundamental parameter in magnetic resonance imaging (MRI), representing the time constant of the exponential decay of transverse magnetization. This decay process occurs as the magnetic moments of protons within a sample lose coherence, leading to a reduction in the detectable MRI signal. Specifically, T2 relaxation time corresponds to the duration when the transverse magnetization decays to approximately 37% of its initial value. This parameter is critical for various MRI techniques, particularly for T2 mapping, which is used to assess tissue properties and detect abnormalities.

Like T1 mapping, T2 mapping employs different pulse sequences designed to measure the T2 relaxation time accurately. These sequences include steady-state free precession (bSSFP), gradient and spin echo (GraSE), and fast spin echo (FSE) acquisitions. Each method offers unique advantages and is chosen based on the specific requirements of the imaging study and the tissues being examined. For instance, GraSE combines the benefits of gradient echo and spin echo sequences, providing efficient T2 mapping with reduced scan times [19].

T2 values, like native T1 values, are influenced by the intrinsic properties of tissues. In the myocardium, T2 values reflect the characteristics of both the intracellular and extracellular compartments. Elevated T2 values typically indicate an increase in free water content within the tissue, making T2 mapping particularly effective for detecting myocardial edema and inflammation. This is crucial in the diagnosis and monitoring of conditions such as myocarditis and myocardial infarction, where tissue water content changes are significant.

The transverse relaxation process, which T2 time quantifies, involves interactions between the magnetic moments of protons within the same molecule. However, in vivo transverse magnetization is affected by local magnetic field inhomogeneities, causing the decay to occur more rapidly than what is predicted by atomic and molecular interactions alone. This faster decay is characterized by the time constant T2*, which is always less than or equal to T2. The presence of magnetic field inhomogeneities can be due to the MRI hardware itself or the presence of paramagnetic materials within the tissue, such as iron, oxygen, and calcium, which distort the magnetic field.

Pulse sequences that emphasize the effects of local magnetic inhomogeneity are known as T2*-weighted sequences. These sequences are particularly useful for detecting the presence of paramagnetic substances like iron and calcium, which can indicate conditions such as hemorrhage or calcifications. T2* imaging is, therefore, a valuable tool in the assessment of myocardial iron loading, as iron’s paramagnetic properties induce local magnetic field distortions.

Compared to T1-mapping techniques, T2-mapping acquisitions present more significant challenges. The increase in T2 values associated with edema is typically small, around 15–20 milliseconds, making it difficult to achieve high signal uniformity. Therefore, precise and consistent imaging techniques are essential to obtain accurate and reliable T2 measurements, ensuring that subtle changes in tissue properties can be detected and appropriately interpreted in clinical practice.

In summary, T2 relaxation time is a critical parameter in MRI that provides insights into tissue properties and abnormalities. The development of various pulse sequences for T2 mapping has enhanced the ability to diagnose and monitor conditions associated with changes in tissue water content and local magnetic field inhomogeneities. The challenges associated with T2 mapping, particularly in achieving high signal uniformity, highlight the need for advanced imaging techniques and careful interpretations of T2 values in clinical settings.

### 3.3. Limitations

Parametric mapping has several applications in a wide spectrum of cardiac pathologies. Owing to its independence from contrast agent administration, this feature renders it particularly appealing for individuals with end-stage renal failure, pediatric patients, pregnant women, or cases necessitating repeated examinations. However, such techniques have some limitations that need to be taken into account [15].

First, the definition of reference values is still a matter of debate. Variability in CMR scanners, in pulse sequences used to create maps (inversion, saturation recovery or hybrid, standard or shortened versions), as well as in patient characteristics such as age and sex, have a deep impact on mapping values. Therefore, it is impossible to establish a universal range of normal values, and it is recommended that each institution determines their own reference values for their scanners, imaging sequence, and contrast protocol. Furthermore, parametric mapping has shown relatively small changes in values among various myocardial disorders, suggesting that it has still a limited discriminatory power and that the final diagnosis should be multiparametric, including other clinical and imaging findings. The value of a single-image voxel results from the contribution of its intracellular, intravascular, and interstitial components. As a result, measurement of an abnormal value cannot identify which specific component is abnormal, which, again, needs clinical interpretation. Finally, the site of measurement may also affect the expected normal values with higher native T1 values at the apical segments compared with the basal and mid ones due to a more pronounced partial volume averaging effect at the apex and lower values at the lateral wall compared with the septum, owing to off-resonance effects and reduced signal-to-noise ratio [15].

## 4. Clinical Applications

### 4.1. Ischemic Heart Disease

The gold standard for the evaluation of ischemic heart disease (IHD) is currently represented by LGE imaging, being able to detect the presence of an ischemic scar, precisely quantify its extension, and identify some other prognostic relevant elements such as microvascular obstruction (MVO) [20]. Complementary to LGE, black-blood T2-weighted IR acquisitions are used for the detection of myocardial edema in the context of acute ischemia [21]. In this setting, T1 and T2 mapping have recently been demonstrated to provide useful information, incremental to conventional LGE and T2-weighted imaging for the evaluation of acute and chronic IHD.

In acute myocardial infarction (AMI), cellular edema occurs very early and can be detected as a prolongation of native T1 and T2 values with high sensitivity and specificity [22]. A CMR examination comprehensive of native T1 mapping, T2-weighted imaging, and LGE performed within 12–48 h after the onset of chest pain has demonstrated a significant relationship between the segmental damaged fraction assessed by either LGE or T2-weighted imaging, and mean segmental T1 values with an overall diagnostic performance of T1 mapping were as good as that of T2-weighted imaging in patients with ST elevation myocardial infarction (STEMI), as well as superior to T2-weighted imaging in patients with non-ST elevation myocardial infarction (NSTEMI) [23]. Beyond the assessment of acute myocardial injury, native T1 mapping has demonstrated prognostic value in predicting left ventricular (LV) remodeling by differentiating reversible and irreversible myocardial injuries.

In the OxAMI Study (Oxford Acute Myocardial Infarction), native T1 mapping performed in STEMI patients acutely (12–96 h post-primary percutaneous coronary intervention) demonstrated an accuracy of 97% in identifying necrotic (irreversibly damaged) vs. edematous myocardium (reversible myocardial injury) when compared with conventional LGE imaging. The T1-defined volume of irreversibly damaged myocardium showed no significant difference with the extent of LGE and strongly correlated with an LV ejection fraction (LVEF) at the 6-month follow-up point [24]. Further early assessments of STEMI patients by native T1 mapping within 2 days post reperfusion showed how T1 values within the hypo-intense infarct core (defined as an area in the center of the infarct territory having a mean T1 ≤ 2 standard deviation of the T1 value of the periphery of the area at risk) are inversely associated with risk of all-cause death or hospitalization for heart failure, regardless of the underlying LVEF [25]. Values of T1 within areas of MVO were found to be slightly higher than those of remote myocardium—but lower than the surrounding infarcted area—and were found to persist up to 2 months after STEMI [24,25]. Of note, studies using post-contrast T1 mapping and the ECV fraction have shown that adverse remodeling of remote myocardium begins within a week of acute myocardial infarction and involves the expansion of the ECV, which progresses proportionally over time to the extent of the ischemic area at risk. The degree of expansion of remote zone ECV at 6 months has been associated with an increase in LV end-diastolic volume and reduction in LVEF [26,27].

In chronic myocardial infarction, the presence of replacement fibrosis can be detected by T1 mapping. However, the overall performance of T1-mapping methods in detecting infarcted areas is modest compared to a visual assessment by LGE [28]. A cut-off value of 42% of the ECV has been proposed for the detection of chronic myocardial infarction scars [29]. The main advantage of native T1 mapping is related to the possibility to differentiate between normal, viable, and non-viable myocardium without the need for contrast agents. In particular, a T1 threshold of ≥1085 ms differentiated viable from non-viable segments with a sensitivity and specificity of 88% in one study at 1.5T [30]. However, it should be noted that long-term remodeling phenomena within the scar, such as lipomatous metaplasia, may shorten T1 values, thus limiting its performance in detecting infarcted scars [31].

### 4.2. Takotsubo Cardiomyopathy

**Figure 3:**
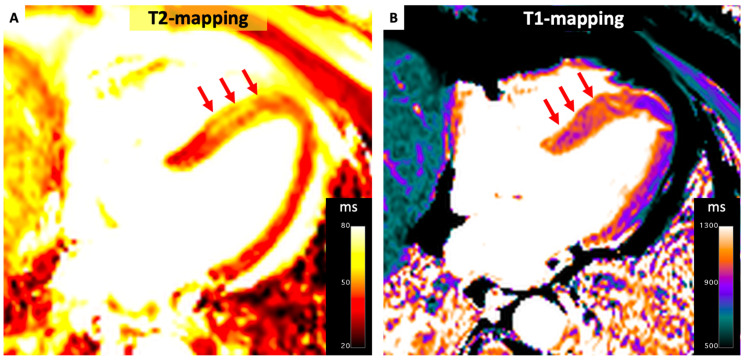
Parametric Mapping in Tako-Tsubo Cardiomyopathy. Case involving a 58-year-old male diagnosed with acute-phase Tako-Tsubo cardiomyopathy, the presentation highlights increase in T2 (panel **A**) and T1 values (panel **B**) across the mid and apical regions of the interventricular septum (red arrows).

Takotsubo (stress-induced) cardiomyopathy is an acute coronary syndrome precipitated by intense emotional or physical stress and characterized by a rapid and spontaneous recovery of LVEF. The classic form involves the preservation of basal segments with akinesis of apical segments, giving the appearance of apical ballooning. However, reverse Takotsubo (with apical sparing and impact on basal segments) can also occur less commonly. The acute phase is characterized by higher native T1 and T2 values for the entire heart (Figure 3) [32]. Interestingly, high native T1 and T2 values can be found even in visually unaffected segments (i.e., basal segments) and are correlated with lower LVEF and prolonged recovery time [32,33]. Data suggest that, despite a rapid normalization of LVEF, myocardial structural changes can persist up to 3–39 months after the event [34,35]. In particular, native T1 values have shown to normalize within 4 months only in segments with unaltered wall motion during the acute phase, while ECV remains abnormal in all LV segments, suggesting a global deposition of interstitial fibrosis [35,36]. Whenever such long-term microstructural alterations have a negative prognostic impact remains unknown, and are important to study as there is a risk of sudden death reported with Takotsubo [37].

### 4.3. Myocarditis

Acute myocarditis (AM) is an inflammatory process of the myocardium accompanied by edema and, hence, by an increase in the free water content of the myocardium, which affects both T1 and T2 relaxation times and, if predominantly extracellular, ECV as well. In the presence of acute inflammation, vasodilation and hyperemia also contribute to the increase in native T1 values [11].

Although endomyocardial biopsy (EMB) remains the gold standard for the diagnosis of AM, it is not frequently performed due to potential serious complications and low sensitivity related to sampling issues with a high false negative rate. For these reasons, CMR is currently the primary diagnostic tool for the assessment of suspected AM [38]. The CMR-based Lake Louise consensus criteria for the diagnosis of AM were initially developed in 2009 and subsequently revised in 2018 [39]. The original criteria were based on signal intensity assessment on conventional T2-weighted sequences (myocardial edema), early gadolinium enhancement (hyperemia), and LGE (necrosis/scar) and had an overall diagnostic accuracy of 83% in identifying AM when ≥2 were present [40]. The development of parametric mapping methods, together with the shortcomings of conventional CMR tissue characterization (i.e., under-detection of diffuse processes), led to a revision of the criteria with the inclusion of T1 and T2 mapping. Parametric mapping allows for a direct quantification of myocardial inflammation without relying on local heterogeneity of signal intensity, thus overcoming some of the limitations of conventional tissue characterization techniques.

**Figure 4:**
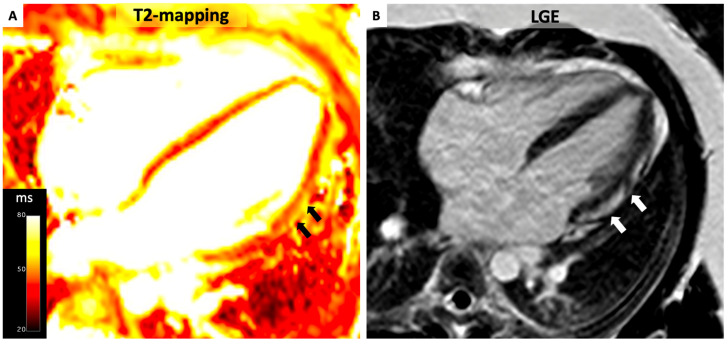
T2 Mapping in Acute Myocarditis. A 31-year-old patient with acute myocarditis affecting the anterolateral left ventricular free wall. Notably, elevated T2 values exceeding 55 ms are evident (Panel **A**, black arrows) indicative of myocardial edema. Additionally, delayed enhancement is observable in the same area (Panel **B**, white arrows).

Several studies have demonstrated the high level of diagnostic accuracy of parametric mapping methods for the diagnosis of AM, being superior to both T2-weighted and LGE imaging (Figure 4) [11,40,41]. In particular, when compared with EMB, native T1 mapping demonstrated the best performance among all CMR parameters in detecting AM, with a diagnostic accuracy of 81% when the CMR evaluation was performed within 14 days of symptom onset [42]. However, T1 mapping may be defective in differentiating between different stages of the disease since there is a substantial overlap between increased T1 values related to inflammation and chronic regional or diffuse fibrosis (DF) as result of the healing process [24,28]. The high sensitivity of T1 mapping makes it very useful in ruling out myocardial abnormalities. However, there is evidence of a higher specificity of T2 mapping for acute inflammation compared to T1. For such reasons, T2 mapping may be particularly useful in ruling active inflammation and discriminating active from healed myocarditis [43]. The expansion of extracellular space can also be detected by ECV [44]. On these bases, the updated Lake Louise criteria combine an edema-sensitive CMR technique (T2-weighted imaging or T2 mapping) together with a T1-based tissue characterization technique to detect non-ischemic myocardial injury (LGE, T1 mapping, or ECV) in order to optimize diagnostic accuracy.

(1) Myocardial edema:
  - Regional/global increase in T2 signal intensity;
  - Regional/global increase in native T2.
(2) Non-ischemic myocardial injury:
  - Regional/global increase in native T1;
  - Regional/global increase in ECV;
  - Regional LGE signal increase.

If at least one criterion in each of the two categories is positive, there is a high chance of AM [11].

### 4.4. Cardiac Sarcoidosis

Sarcoidosis is a systemic inflammatory disease characterized by lymphocyte CD4+-mediated formation of non-necrotizing granulomas. Cardiac involvement is relatively rare and can lead to a wide spectrum of clinical manifestations ranging from potentially reversible conduction disorders and atrial arrhythmias to life-threatening ventricular arrhythmias (VA) and severe heart failure [45]. Myocardial damage is driven by active inflammation with myocyte loss and reparative fibrosis that may progress to biventricular dilation and dysfunction, as well as refractory arrhythmias [46,47,48].

The utility of LGE in diagnosis and staging of cardiac involvement is well known, with the presence of LGE being documented in up to one quarter of patients with systemic disease [49]. Recently, studies have shown how subclinical cardiac involvement can be identified, even in patients with normal LVEF and without evidence of LGE, by higher values of T1 and T2 and expanded ECV compared to healthy controls [50]. However, while the presence of LGE strongly correlates with adverse clinical outcomes, the clinical impact of abnormal parametric findings remains to be elucidated. Another promising application of parametric mapping in this context relies on the possibility to longitudinally evaluate treatment response, with the suppression of inflammation being one of the major determinants of long-term outcomes [48]. Subjects effectively treated with immune suppression showed a significant reduction in peak myocardial T2 values (average 15% reduction) paralleling with objective clinical improvement [51,52].

### 4.5. Autoimmune Disorders

Parametric mapping can be really helpful to detect inflammation and interstitial fibrosis, even in patients with no overt evidence of cardiac involvement, in the context of inflammatory disorders such as rheumatoid arthritis, systemic sclerosis, and systemic lupus erythematosus [39]. In patients with rheumatoid arthritis, as well as in patients with systemic sclerosis, focal areas of LGE could be identified in 55% of them. Compared to healthy controls, affected patients typically present higher native T1, T2 and ECV values, which has been correlated with disease activity and severity [53,54,55]. In lupus erythematosus, increased T2 values have been correlated with disease activity, and both native T1 and T2 mapping may be used to monitor the response to anti-inflammatory treatment [56,57,58].

T2 mapping has proven to be a crucial imaging technique for assessing myocardial involvement in patients with systemic lupus erythematosus (SLE), which can be present in up to 9% of cases, with a higher prevalence in younger patients and females. Notably, myocarditis is found in 50–80% of autopsied SLE patients, indicating a significant burden of subclinical myocardial involvement.

Studies have found increased T2-weighted imaging (T2WI) signals in active SLE patients compared to healthy controls, indicating myocardial edema and inflammation. However, the clinical application of black-blood T2WI is limited by inconsistent imaging quality due to various factors such as coil sensitivity and signal loss. The interpretation of edema via T2WI relies on subjective evaluation, prompting the use of the T2 ratio method for semi-quantitative assessments, with a ratio greater than 1.9 defining global myocardial edema. Yet, this method may be unreliable in SLE patients due to confounding factors like myositis [59]. On the other hand, T2 mapping provides reliable and quantitative myocardial T2 values, enhancing sensitivity in detecting myocardial inflammation. Research has shown a correlation between SLE disease activity and T2 relaxation times, with longer T2 values in SLE patients compared to controls (58.2 ± 5.6 ms vs. 52.8 ± 4.4 ms) [60]. A multivendor observational study involving 92 SLE patients revealed a correlation between myocardial edema detected by T2 mapping and highly sensitive troponin levels [58].

Furthermore, T2 mapping is valuable for monitoring the efficacy of therapeutic interventions. Studies have demonstrated that SLE patients undergoing immunosuppressive therapy showed a significant reduction in myocardial T2 values, correlating with clinical improvement and reduced myocardial inflammation. This demonstrates the potential of T2 mapping not only for initial diagnosis but also for tracking disease progression and treatment response [56].

### 4.6. Transplant Rejection

The gold standard to detect acute cardiac allograft rejection (ACAR) is represented by EMB. Nonetheless, in the last few years, myocardial CMR mapping has emerged as a promising non-invasive alternative. In a study comparing CMR to EBM in 20 transplant recipients, patients with ACAR had significantly higher T2 values, as well as ECV, compared to patients with no rejection. In particular, a T2 threshold of ≥58 ms showed a diagnostic accuracy of 90%, and an ECV cut-off value of ≥32% showed an accuracy of 85% [61]. Another study including 58 transplant recipients undergoing repeated CMR evaluation and EMB showed that myocardial T2 was significantly higher in both patients with biopsy-proven ACAR (52 ± 5 ms) and in patients with a past history of ACAR (51 ± 4 ms) compared to transplant recipients with no history of ACAR (49 ± 4 ms) and control subjects (45 ± 2 ms). However, no significant difference was observed between patients with active ACAR and those with a past history of ACAR. Of note, ECV was higher in patients with active ACAR (32 ± 4%) compared to those without active ACAR, regardless of a previous history of ACAR (no history of ACAR: 27 ± 3%; past history of ACAR: 27 ± 4%), thus showing how a combination of CMR parametric mapping parameters may be able to identify active ACAR [62]. In a recent prospective randomized study, the findings from 401 CMR studies and 354 EMB procedures in 106 participants showed that CMR-based assessment was highly accurate in detecting ACAR, demonstrating high sensitivity, specificity, and negative predictive value (area under the curve, 0.92; sensitivity, 93%; specificity, 92%; negative predictive value, 99%). High-grade rejection rates were similar between the CMR and EMB groups. However, the CMR group benefited from significantly lower rates of hospitalization and infection, suggesting a potentially safer and less invasive surveillance method [63].

### 4.7. Non-Ischemic Dilated Cardiomyopathy

In patients with non-ischemic dilated cardiomyopathy (NIDCM), the presence, amount, and distribution of fibrosis detected by LGE-CMR has convincingly demonstrated a substantial impact on the lack of response to guideline-directed medical therapy, long-term mortality, and risk of major VA [64]. However, up to 70% of patients with NIDCM do not have any definite evidence of scars detectable by LGE [65]. Since collagen deposition in NIDCM is often diffuse, LGE imaging may fail to show fibrosis, as its detection is mostly qualitative and relies on the spatial heterogeneity of signal intensity (i.e., difference in signal intensity between scarred and normal adjacent myocardium) [1]. In this context, parametric mapping techniques such as T1 mapping may help to detect DF. Typically, native T1 and ECV increase while post-contrast T1 decreases. Native and post-contrast T1 mapping have been correlated with EMB-proven collagen volume fraction and extracellular space fraction in NIDCM patients [66]. Significantly abnormal T1 times have also been shown in NIDCM patients presenting with VA compared to those without VA, with T1 time being an independent predictor of VA beyond LVEF and LGE [67]. Calculating the T1 time from a single septal sampling site has been demonstrated to represent a reliable marker of DF burden [1,66,68]. In a mixed cohort of NIDCM and ischemic patients, T1 times were independently associated with appropriate ICD therapy at follow-up [68]. Similarly, in patients with NIDCM, septal T1 times have been independently associated with a 10% increase in all-cause mortality, as well as a 12% increase in appropriate ICD therapies per each 10 ms variation [1]. In a single-center study of 115 NIDCM patients eligible for the implantation of primary prevention ICDs, global native T1 (mean value calculated on five short-axis slices) showed a comparable accuracy rate (C-statistic: 0.76) to the presence, location, and extent of LGE in predicting the primary endpoint of appropriate ICD therapy and sudden cardiac death [69]. These data are supported by a recent meta-analysis of eight studies including a total of 1242 patients. Both ECV (HR 1.38; 95% CI 1.18–1.61) and native T1 time (HR 1.20; 95% CI 1.14–1.27) were independently correlated with mortality and major adverse cardiovascular events (MACEs) during follow-up [70]. The utility of routine ICD implantation in patients with NIDCM and LVEF below 35% has recently been questioned [71]. It is possible that in the near future, risk stratification refinement with CMR, including both LGE and parametric mapping techniques, will modify the current indication to the implantation of primary prevention ICDs [72].

T2 mapping in cardiovascular magnetic resonance (CMR) has demonstrated significant potential in evaluating myocardial changes in heart failure patients. Elevated T2 values indicate myocardial edema, common in various inflammatory cardiomyopathies, and provide substantial utility in differentiating between myocarditis and non-ischemic dilated cardiomyopathy (NIDCM). Patients with heart failure (HF) and moderately reduced LVEF exhibit longer global left ventricular T2 time compared to healthy controls, a trend also observed in HF with preserved EF (HFpEF) [73]. The global myocardial T2 relaxation time correlates significantly with various clinical parameters in these patients, including quality of life, 6 min walking test, glomerular filtration rate, and N-terminal pro-brain natriuretic peptide (NT-proBNP) levels [73]. Notably, global myocardial T2 relaxation time may serve as an indicator of compensation in HF, with acute decompensated HF characterized by prolonged T2 time, which shortens after decongestion. This susceptibility to edema suggests that myocardial tissue may be more vulnerable to fluid accumulation due to the dynamic nature of venous and lymphatic flow during the cardiac cycle [74]. In a study of patients with recent-onset heart failure, those with biopsy-confirmed active myocarditis had significantly elevated global myocardial T2 values compared to those with normal biopsies. A global myocardial T2 value of ≥60 ms was identified as the optimal threshold for detecting active myocarditis, supporting patient stratification for further invasive diagnostic procedures such as EMB [75]. Additionally, parametric mapping has been assessed for the non-invasive detection of chronic myocardial inflammation in patients with heart failure with reduced ejection fraction (HFrEF). Among 52 HFrEF patients, 33 had EMB-detected myocardial inflammation. Global T1 and T2 values were significantly higher in HFrEF patients compared to healthy controls (T1: 1275 ± 69 ms vs. 1175 ± 44 ms, *p* < 0.001; T2: 40.0 ± 3.4 ms vs. 37.9 ± 1.6 ms, *p* < 0.001). However, no significant differences in T1 and T2 values were observed between inflammation-positive and inflammation-negative HFrEF patients. Thus, while T1 and T2 mapping correlated with HFrEF prevalence, they did not effectively distinguish chronic myocardial inflammation, suggesting that EMB remains the preferred method for detecting such inflammation in HFrEF patients [76].

### 4.8. Left Ventricular Non-Compaction

Left ventricular noncompaction (LVNC) is a rare cardiomyopathy resulting from an arrest of the normal embryogenetic development of the myocardium, leading to the formation of prominent trabeculation with deep intertrabecular recesses of the LV wall. Common manifestations of the disease are heart failure, VA, and thromboembolic events [77]. Small-scale studies have found elevated native T1 values in non-compacted segments even when LGE is absent, suggesting that native T1 mapping can detect the presence of fibrosis early in the disease course before any clear evidence of LGE [78]. Both native T1 and ECV were higher in LVNC compared to healthy controls, but ECV seemed to have a greater clinical value, being independently correlated with LV function and the risk of VA [79,80].

### 4.9. Hypertrophic Cardiomyopathy

Shorter post-contrast T1 times and higher ECV have been repeatedly found in patients affected by hypertrophic cardiomyopathy (HCM) because of myocardial fiber disarray and interstitial collagen deposition. In a study comparing HCM patients with sarcomeric gene mutations to gene carriers without evidence of hypertrophy and healthy subjects, ECV appeared to be increased in both gene+/phenotype+ (mean ECV, 36%) and gene+/phenotype- (mean ECV, 33%) patients compared to healthy controls (mean ECV, 27%), while areas of LGE were present in more than 60% of genotype+/phenotype patients but in none of the phenotype-/genotype- patients, suggesting early fibrotic remodeling preceding an overt manifestation of the disease [81]. Significant increases in native T1 and ECV values suggestive of DF have been observed in hypertrophied segments, even in the absence of regionally apparent LGE and LV outflow tract obstruction, and they correlated with wall thickness and disease severity [78]. From a prognostic perspective, in a single-center cohort of 263 HCM patients, native T1 and ECV were independently correlated with the 28-month risk of MACEs, including cardiac death, heart transplant, aborted sudden death, and cardiopulmonary resuscitation after syncope. In particular, every 3% increase in ECV was associated with a 1.4-fold increase risk of MACEs [82].

An interesting application of mapping techniques is the possibility to differentiate pathologic hypertrophy from physiologic adaptation to intense training, as in athlete’s heart. In athlete’s heart, LV hypertrophy is a consequence of increased myocardial cellular mass rather than extracellular fraction, as demonstrated by the increase in LV mass according to training intensity with non-significant changes in ECV [83]. Of note, ECV may be lower than normal in athlete’s heart in comparison to sedentary controls and HCM patients, owing to the increases in cellular mass [83,84].

### 4.10. Cardiac Amyloidosis

Amyloidosis is a systemic infiltrative disorder characterized by the deposition of misfolded proteins (amyloids) in the interstitial space of several organs. Heart involvement is frequent and characterized by a hypertrophic phenotype manifesting as heart failure with preserved ejection fraction in the initial phases of the disease progressively evolving through LV disfunction in advanced stages. There are two types of cardiac amyloidosis: the more prevalent one related to immunoglobulin light-chain-derived (AL) amyloids and the other to transthyretin (ATTR) amyloids. ATTR is further subdivided into genetic (hereditary ATTR) vs. wild-type ATTR. Regardless of the specific type of amyloidosis, proteins misfold, forming insoluble fibrils that accumulate in the interstitial space at a systemic level. The gold standard for the detection of cardiac involvement is represented by EMB, which, however, may be suboptimal due to mis-sampling. Delayed enhancement CMR is a valuable diagnostic tool [85]. Common findings range from a diffuse subendocardial tramline pattern with early blood-pool darkening on Look Locker scout images to transmural enhancement at later stages. A peculiar finding is related to the characteristic diffuse pattern of LGE, which makes the nulling of normal myocardium particularly difficult [86].

**Figure 5:**
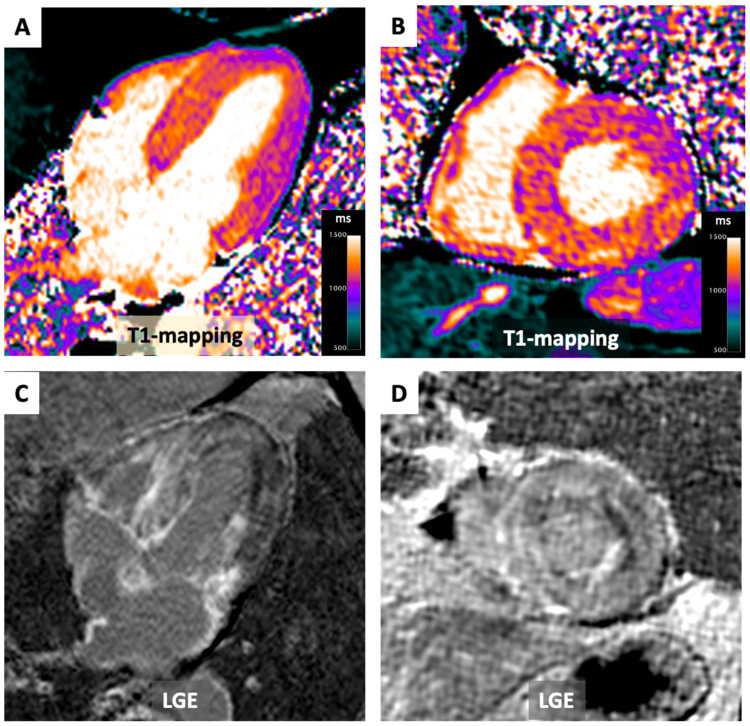
T1 Mapping in Cardiac Amyloidosis. A 76-year-old patient diagnosed with transthyretin cardiac amyloidosis. Noteworthy findings encompass severe left ventricular concentric hypertrophy with T1 values surpassing 1100 ms. (Panel **A**: long axis 4-chamber view and Panel **B**: mid-ventricular short axis view). Furthermore, global myocardial enhancement is apparent in the LGE (late gadolinium enhancement) images (Panels **C**,**D**), displaying intensity akin to that of the blood pool.

Native T1 has demonstrated great value in the diagnosis and prognosis of patients affected by cardiac amyloidosis. In both AL and ATTR amyloids, T1 values are extremely elevated compared to all other cardiac diseases, with mean values in the range of 1140 ± 61 ms (Figure 5) [12]. Native T1 appears to be elevated, even in early stages of the disease when there is no clear evidence of LGE yet. A native T1 value of ≥1020 ms has been proposed as a cut-off value to identify cardiac involvement in patients with systemic amyloids, and it has demonstrated a diagnostic accuracy of 92% [12]. The interstitial expansion resulting from amyloid deposition makes ECV a surrogate of amyloid burden with prognostic value. In particular, ECV in the range of 48–55% has been correlated with an independent 4-fold increased risk of death in both patients with AL and ATTR amyloidosis [87]. ECV can be also applied in monitoring treatment response [88,89]. Subtle differences can be found in AL vs. ATTR amyloidosis, with a higher ECV in ATTR and a higher native T1 in AL [90]. Nonetheless, given the substantial overlap between AL and ATTR amyloidosis and the therapeutic implications of the differential diagnosis (liver transplantation, novel TTR specific treatment in ATTR vs. chemotherapy, autologous stem cell transplantation in AL), further testing is required to reliably distinguish between the two types.

### 4.11. Anderson–Fabry Disease

**Figure 6:**
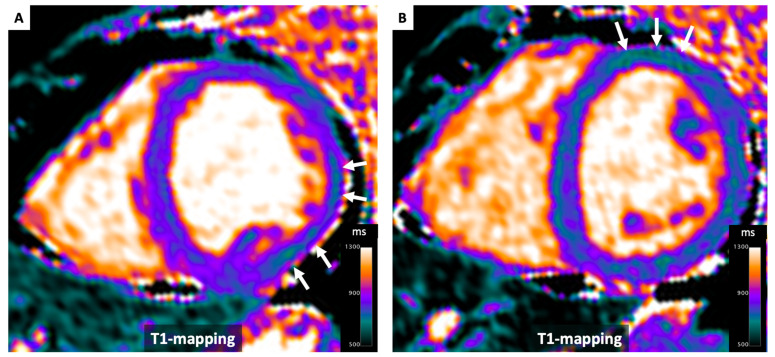
T1 Mapping in Fabry’s disease. A case involving a 40-year-old female patient with a confirmed diagnosis of Anderson-Fabry disease. Notably, T1 values below 900 ms are evident in the left ventricular free wall (Panels **A**,**B**), even in the absence of overt left ventricular hypertrophy (white arrows). This case effectively demonstrates how T1 mapping can serve as a valuable tool for the early detection of cardiac involvement in this condition.

Anderson–Fabry disease (AFD) is an X-linked lysosomal storage disease characterized by a deficiency in alpha-galactosidase A activity, resulting in a progressive accumulation of intracellular lipids (globotriaosylceramide within lysosomes) and causing multi-organ dysfunction. Cardiac involvement is characterized by LV hypertrophy, heart failure, and arrhythmias [91]. As a result of the intra-cellular accumulation of glycosphingolipids, native T1 is typically low (<900 ms) (Figure 6). Interestingly, T1 values appear to be lower than the normal range, even in the absence of overt LV hypertrophy, making T1 mapping a useful tool for the early detection of subclinical cardiac involvement [13]. The decrease in native T1 values becomes more severe as LV hypertrophy evolves [13]. Therefore, T1 mapping allows for the differentiation of Fabry disease from other causes of LV hypertrophy. In advanced stages of the disease, mid-myocardial LGE may appear in the inferolateral wall of the LV because of a fibrotic process. As a consequence, native T1 in this area may appear pseudo-normalized or even elevated [13]. Conversely, ECV is typically normal because of the primarily intracellular involvement of the disease [92]. CMR assessment allows for the precise tracking of myocardial changes associated with Fabry disease, offering insights into treatment response that traditional methods like echocardiography cannot provide. CMR not only measures left ventricular mass accurately but also tracks intracellular sphingolipid accumulation using T1 mapping and identifies myocardial inflammation and scars with T2 mapping and late gadolinium enhancement (LGE) imaging. Enzyme replacement therapy appears to slow sphingolipid storage but may not entirely prevent toxic myocardial effects. Future research is needed to evaluate the long-term effects of therapy. Establishing treatment thresholds based on CMR parameters such as native T1 or T2 times is essential for effectively guiding therapy. Rigorous protocols for CMR imaging will enable the monitoring of myocardial treatment response. Overall, CMR holds promise in improving outcomes and quality of life for patients with Fabry disease by guiding personalized therapy [93].

### 4.12. Iron Overload

Myocardial iron overload (MIO) can be primarily genetic, as in hemochromatosis, or may be secondary to repeated blood transfusion such as in cases of thalassemia major (TM). CMR is particularly useful to detect iron overload, as iron is a paramagnetic element that alters the magnetic field, shortening T1, T2, and T2*. In particular, T2* values inversely correlate with iron overload [94]. Low T1 values have been shown in iron overload cardiomyopathy, but, even if more reproducible, T1 is less pathologically specific, and its increase in cases of expansion of extracellular volume may mask iron deposition, especially in early stages, thus making T2* the gold-standard method to quantify iron deposition [14]. The mapping of T2* can also be used to monitor disease progression and therapy. Serial CMR assessment has been proven to play a role in the modulation of iron chelation therapy, with a significant impact on clinical outcomes [95].

In TM, parametric mapping can detect subtle myocardial structural abnormalities even in a subclinical setting, stage the severity of the disease, and monitor its progression. A study investigated the diagnostic performance of native T1 and T2 mapping for MIO in TM patients. Among 200 patients undergoing CMR, 8 (4.0%) were diagnosed with MIO. Significant differences in native T1 and T2 times were observed across patients with varying degrees of MIO. Optimal cut-off values for detecting MIO were 887 ms for T1 and 52 ms for T2, with native T1 mapping demonstrating superior sensitivity, specificity, and area under the curve [96]. Another study assessed the utility of extracellular volume (ECV) in TM patients, finding that global ECV values were higher in females and significantly elevated in patients with more advanced stages of MIO. Patients with reduced global heart T2 values exhibited higher ECV values, potentially indicating diffuse interstitial fibrosis. Additionally, patients with a history of heart failure had significantly higher ECV values, suggesting that increased ECV in TM patients may be linked to both MIO and heart failure [97].

### 4.13. Chemotherapy

Increased ECV and native T1 values have been reported in patients exposed to anthracyclines even before overt LV dysfunction occurred, proving how CMR can reliably identify subtle changes in myocardial structure and detect early cardiotoxicity, making it particularly useful in the serial monitoring of chemotherapy-induced cardiotoxicity [98]. Of note, T2 mapping may identify myocardial edema as an initial and reversible stage of anthracycline-induced cardiotoxicity, allowing for treatment modulation [99].

### 4.14. Valvular Heart Disease

In valvular heart disease, together with the progression of valvular abnormality, the heart muscle also undergoes progressive deterioration, reflecting structural changes secondary to pressure/volume overload [100]. Parametric mapping has been proposed to detect early subclinical structural abnormalities of the myocardium and guide therapy. In patients with both aortic stenosis (AS) or aortic regurgitation (AR), native T1 and ECV showed a good correlation with interstitial fibrosis on EMB, suggesting a maladaptive response to overload [101]. In severe AS, native T1 values appear to be higher, even in asymptomatic patients with preserved LVEF, and further increased in symptomatic patients [102]. Similar findings have been described in severe AR, opening the way for a possible role of CMR mapping techniques in the optimization of surgical timing [103]. Only few data are currently available on the topics of mitral valve disease showing similar trends to higher ECV in asymptomatic patients with moderate to severe MR and higher T1 values in patients with mitral valve prolapse, regardless of the degree of valvular dysfunction [104].

### 4.15. Future Applications

The future of CMR parametric mapping hinges on achieving standardization, broadening clinical applications, and advancing acquisition and reconstruction techniques to facilitate thorough, quantitative, and contrast-free tissue characterization. Within this framework, techniques like SMART (Simultaneous Multi-slice Accelerated Reconstruction for Tissue) allow for the simultaneous acquisition and reconstruction of co-registered CMR images with different contrast weightings, employing sparse sampling principles to significantly reduce scan times. Furthermore, advancements in 3D mapping techniques and spatio-temporal parallel imaging enable whole-heart coverage at higher spatial resolutions, surpassing the limitations of traditional 2D multi-slice approaches. These innovations are poised to propel CMR parametric mapping into a pivotal role in cardiovascular diagnostics [105,106].

There are several ongoing areas of the potential development of parametric mapping techniques. One of these is myocardial perfusion, in which the measurement of T1 reactivity reflects changes in myocardial T1 values before and after pharmacological vasodilatation, allowing for the detection for coronary artery disease (CAD) and, possibly, the differentiation of myocardial ischemia due to obstructive epicardial CAD from microvascular dysfunction [107]. Normal myocardium shows an increase of about 6% of T1 values during vasodilatory stress compared to chronic infarcted myocardium, which has significantly elevated T1 values at rest with no change during stress [107]. In the presence of significant CAD, mildly elevated T1 values are detected at rest with no significant changes during stress due to a demand/supply imbalance. In patients with type II diabetes without significant CAD, adenosine stress shows a blunted response in T1 values, likely reflecting microvascular dysfunction [108]. In severe AS with LV hypertrophy and without CAD, there is a substantial reduction in T1 reactivity during adenosine stress compared with controls, which normalizes in about seven months after aortic valve replacement [109].

While LGE plays a protagonist role in the substrate characterization of cardiac arrhythmias, with particular regard to VA, a growing body of evidence has demonstrated its impact in procedural planning in patients undergoing catheter ablation, yet there is little evidence demonstrating a possible role for T1 mapping, especially in cases without clear evidence of dense scars [110]. In particular, in patients with NIDCM undergoing VT ablation, there is some initial evidence that T1 mapping may be of incremental value to disclose concealed myocardial substrate, with a significant association between the burden of DF quantified by T1 mapping and the extent of an abnormal electrical substrate determined by invasive voltage mapping [3,80].

## 5. Conclusions

Parametric CMR mapping techniques offer the distinct advantage of directly quantifying even subtle or diffuse myocardial abnormalities. This eliminates the reliance on spatial heterogeneities in signal intensity and obviates the necessity for contrast agents. This attribute holds significant clinical value, as it will enhance the accuracy of diagnosing and stratifying risks associated with various cardiac disorders.

## Data Availability

Not applicable.
